# Safety and efficacy of genomic biomarker-guided neoadjuvant therapy for locally advanced and oligometastatic prostate cancer (SEGNO): study protocol for an open-label prospective phase II umbrella clinical trial

**DOI:** 10.1186/s12885-025-13826-5

**Published:** 2025-03-10

**Authors:** Haichao Huang, Tao Wang, Wei Li, Zhun Wu, Xuegang Wang, Jinchun Xing, Bin Chen, Kaiyan Zhang

**Affiliations:** 1 Department of Urology, School of Medicine, The First Affiliated Hospital of Xiamen University, Xiamen University, 55 Zhenhai Road, Siming District, Xiamen, 361003 Fujian China; 2 The School of Clinical Medicine, Fujian Medical University, Fuzhou, 350122 Fujian China

**Keywords:** Genomic biomarker-guided, Neoadjuvant therapy, Locally advanced, Oligometastatic, Prostate cancer

## Abstract

**Background:**

The management of locally advanced prostate cancer (PCa) and oligometastatic prostate cancer (OMPCa) remains a clinical challenge. The heterogeneous nature of PCa prompts a need for precision treatment. This study aims to verify whether genomic biomarker-guided neoadjuvant therapy for locally advanced PCa and OMPCa can result in an improvement in the pathological responses and survival outcomes in a Chinese population.

**Methods:**

In this open-label prospective phase II umbrella clinical trial, 40 patients will be enrolled. Next-generation sequencing data analysis of PCa tissues from the diagnostic needle biopsies will be performed. The genomically evaluable patients will be divided into 4 groups on the basis of genomic testing results, and receive 6 cycles of patient-tailored neoadjuvant systemic therapy targeted to alternative molecular pathways (including parmiparib, cisplatin, tislelizumab or docetaxel, respectively), and both in combination with rezvilutamide and goserelin microspheres. The primary endpoint is the rate of pathologic complete response. Secondary endpoints include rates of clinical complete response and pathological minimal residual disease (defined as residual tumor 5 mm or less), overall survival, progression-free survival and safety outcomes.

**Discussion:**

SEGNO, to the best of our knowledge, is the first umbrella clinical trial designed to provide high-level evidence to support the implementation of genomic biomarker-guided neoadjuvant therapy for locally advanced PCa and OMPCa.

**Trial registration:**

Clinicaltrial.gov, NCT06387056.

**Supplementary information:**

The online version contains supplementary material available at 10.1186/s12885-025-13826-5.

## Background

Prostate cancer (PCa) represents the second most commonly diagnosed malignance in men worldwide [1]. Of all newly diagnosed patients with PCa in developing Asian counties, more than 50% present with advanced-stage disease and have unfavorable survival outcomes, as opposed to early-stage identification common in Western countries [2]. A multi-modal treatment strategy, which included local surgical treatment and neoadjuvant systemic therapy, has been reported in an attempt to achieve pathological response and increase survival outcomes in patients with locally advanced PCa [3–6] and oligometastatic prostate cancer (OMPCa) [7]. However, no consensus yet exists regarding the standard neoadjuvant therapy (NT) regimen.

Neoadjuvant systemic therapy is defined as an approach administered prior to local surgical treatment in an attempt to decrease local tumor burden and treat possible micrometastases present at the time of local therapy [8]. This strategy before radical prostatectomy (RP) for high-risk PCa has been extensively explored. The mechanism of neoadjuvant hormonal therapy (NHT) is to target the androgen receptor (AR) axis. However, in studies evaluating the efficacy of conventional androgen deprivation therapy (ADT) for high-risk PCa, no survival benefit was observed. Inability to completely block the production of intratumoural tissue and adrenal androgens, which can still activate the AR, was considered to be the main cause of the low pathological response rate and lack of improvement in survival outcomes of conventional ADT [9–10]. However, despite novel androgen receptor signaling inhibitors (ARSIs), including abiraterone, enzalutamide and apalutamide, were used in combination with conventional ADT to achieve potent targeting of AR axis, limited improvement in terms of pathological complete response (pCR) rate was observed [11–14]. Comprehensive molecular analyses of primary prostate cancer showed that pre-existing castration-resistant cells may be an early event and were selected for by NHT targeting the AR axis [15]. This finding was suggested to be responsible for the limited improvement in pathological outcomes of NHT and facilitated the addition of chemotherapy to neoadjuvant setting. With the advancement of next-generation sequencing (NGS) technology, actionable PCa targets other than the AR have been revealed, meaning that PCa is a complex disease with significant heterogeneity. Thus, precision treatment, also known as personalized medicine, which can tailor treatment to the individual patient based on their specific tumor molecular characteristics is urgently needed. The value of precision treatment in the neoadjuvant setting for high-risk PCa is currently under investigation [16].

Despite several studies have investigated the role of neoadjuvant systemic therapy for locally advanced PCa and OMPCa, there is currently no optimal management in this setting. In a phase II study with neoadjuvant docetaxel chemotherapy and complete androgen blockade in locally advanced and high-risk PCa, no pCR was revealed in postoperative pathological analyses [17]. In a retrospective comparative study evaluating neoadjuvant chemo-hormonal therapy (NCHT) for very high risk locally advanced PCa, 9 (9/52, 17.3%) and 6 (6/70, 8.5%) patients revealed undetectable tumors after NCHT and NHT, respectively, and it is important to note that no pCR was revealed in non-NT group [4]. In another prospective randomized trial evaluating the benefits of docetaxel-based NCHT for locally advanced PCa, only 1 (1/83, 1.2%) patient achieved pCR after 6 cycles of NCHT, whereas no patient was found to achieved pCR in NHT group [6]. In a prospective study evaluating NCHT for OMPCa, 2 (2/17,11.7%) patients had pCR after 6 cycles of NCHT [7]. Several trials have also investigated the use of ARSIs, either as monotherapy or in combination, in the neoadjuvant setting; however, the outcomes have been less promising than anticipated. Montgomery et al. reported that none of the patients receiving neoadjuvant enzalutamide monotherapy achieved a pCR or pathological minimal residual disease (pMRD) < 3 mm. In contrast, among patients treated with enzalutamide in combination with dutasteride and leuprolide, 1 of 23 (4.3%) achieved pCR and 3 of 23 (13.0%) achieved pMRD [12]. Similarly, in the NEAR trial (a Phase II study evaluating apalutamide monotherapy in 30 patients), none of the patients achieved pCR [18]. More recently, McKay et al. conducted a Phase II randomized controlled trial comparing 6 months of neoadjuvant abiraterone plus enzalutamide and ADT (*n* = 50) versus enzalutamide plus ADT alone (*n* = 25) [11]. The pCR rates were 10% and 8%, respectively, indicating that the addition of a second ARSI did not substantially improve pathological responses over enzalutamide plus ADT alone. Hence, the management of locally advanced PCa and OMPCa remains a clinical challenge, which prompts a need for precision treatment. In a recent investigation of Chi and colleagues, 4 (4/14, 28.6%) locally advanced PCa patients with germline DNA damage repair gene alterations achieved pCR after the administration of neoadjuvant docetaxel plus cisplatin chemo-hormonal therapy [3]. This finding suggested a potential role of precision treatment in improving the clinical outcomes of locally advanced PCa and OMPCa. However, research of precision cancer treatment emphasis on locally advanced PCa and OMPCa is still limited, especially in the Asian population.

Based on the above findings, most studies have utilized pCR as a primary endpoint. Furthermore, in neoadjuvant trials for early-stage breast [19] and lung cancers [20, 21], pCR and major pathologic response (MPR; also referred to as pMRD) have demonstrated a strong correlation with event-free survival (EFS) and overall survival (OS). Although some controversies remain [22], these measures are now widely recognized as surrogate endpoints for OS in numerous tumor types, including prostate cancer. A comprehensive pooled analysis of contemporary neoadjuvant clinical trials encompassing 72 patients demonstrated that patients achieving either pCR or pMRD showed no incidence of PSA recurrence, thereby underscoring a significant association between these pathological responses and favorable clinical outcomes [23]. This observation was subsequently corroborated by an expanded cohort analysis (*n* = 117), which further validated that pCR/pMRD status remained a significant predictor of PSA failure-free survival, thereby strengthening their utility as reliable prognostic indicators [24].

This study aims to evaluate the pathological responses, survival benefits and safety profiles of patients with locally advanced PCa and OMPCa treated with patient-tailored neoadjuvant systemic therapy on the basis of NGS genomic profiling results in a Chinese population.

## Methods and study design

### Study design

This open-label prospective phase II umbrella clinical tria will investigate the safety and efficacy of genomic biomarker-guided NT for locally advanced and oligometastatic prostate cancer. This study will be conducted at the First Affiliated Hospital of Xiamen University, Xiamen, China. The planned duration of recruitment for the study is from April 2024 to April 2026.

### Eligibility criteria

The inclusion and exclusion criteria for the study are listed in Box 1.

### Trial intervention

#### Baseline evaluation

**Fig. 1:**
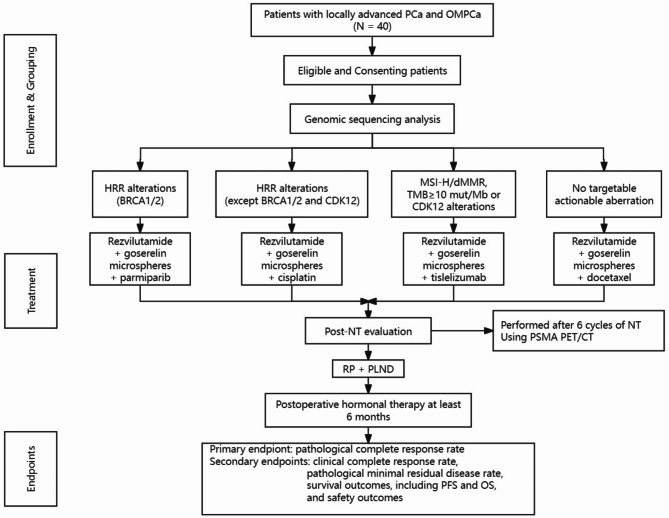
Study flow chart describing the process of enrollment, grouping, treatment and endpoints. PCa, prostate cancer; OMPCa, oligometastatic prostate cancer; HRR, homologous recombination repair; MSI-H, microsatellite instability-high; dMMR, mismatch repair–deficient; TMB, tumor mutational burden; NT, neoadjuvant therapy; PSMA PET/CT, prostate-specific membrane antigen positron emission tomography/ computed tomography; RP, radical prostatectomy; PLND, pelvic lymph node dissection; PFS, progression-free survival; OS, overall survival

Patients with histologically confirmed locally advanced PCa and OMPCa who are eligible for this study will be evaluated for baseline characteristics. NGS data analysis of PCa tissues from the diagnostic needle biopsies will be performed. A study flow chart is shown in Fig. 1.

#### Patient-tailored neoadjuvant systemic therapy

Each participant will be treated with rezvilutamide (240 mg PO QD) plus goserelin microspheres (3.6 mg IM Q4W), while genome sequence analysis is being done. Then, according to the results of the genomic profile, participants (*N* = 40) will be assigned to 4 groups (no less than 5 cases per group) receiving 6 cycles of patient-tailored NT targeted to alternative molecular pathways, and both in combination with rezvilutamide and goserelin microspheres: Arm 1: Homologous recombination repair (HRR) alterations (BRCA1/2) (Rezvilutamide: 240 mg PO QD + goserelin microspheres: 3.6 mg IM Q4W + parmiparib: 60 mg PO QD); Arm 2: HRR alterations (except BRCA1/2 and CDK12) (Rezvilutamide: 240 mg PO QD + goserelin microspheres: 3.6 mg IM Q4W + cisplatin: 70 mg/m^2^ IV Q3W); Arm 3: Microsatellite instability-high (MSI-H), mismatch repair–deficient (dMMR), tumor mutational burden (TMB) ≥ 10 mut/Mb or CDK12 alterations without other HRR alterations (Rezvilutamide: 240 mg PO QD + goserelin microspheres: 3.6 mg IM Q4W + tislelizumab: 200 mg IV Q3W); Arm 4: No targetable actionable aberration (Rezvilutamide: 240 mg PO QD + goserelin microspheres: 3.6 mg IM Q4W + docetaxel: 70 mg/m^2^ IV Q3W).

#### Post-NT evaluation and treatment

A following prostate-specific membrane antigen positron emission tomography/ computed tomography (PSMA PET/CT) will be performed after 6 cycles of NT to evaluate the rate of clinical complete response (cCR). RP plus pelvic lymph node dissection (PLND) will follow the NT, and pathological response (pCR and pMRD) will be evaluated.

#### Postoperative treatment

All participants will receive long-term post-operative hormonal therapy (rezvilutamide plus goserelin microspheres) for at least 6 months. Participants will be followed up every 3 months for the first year, every 6 months for 2–3 years, and annually thereafter, including monitoring of serum PSA, testosterone levels, and treatment-related complications. When disease progression is suspected, prostate multi-parameter magnetic resonance imaging, whole body bone scan or PSMA PET/CT will be further evaluated, and salvage treatments will be performed in accordance with standard clinical practice.

### Study endpoints

#### Primary endpoint

The primary outcome will be the rate of pCR, which is defined as the absence of disease in all removed specimen including the prostate and lymph nodes under pathologic examination [25].

#### Secondary endpoints

Secondary endpoints will include: (1) the rate of pMRD, defined as residual tumor 5 mm or less), (2) the rate of cCR, defined as undetectable disease in perioperative PSMA PET/CT, (3) survival outcomes, including OS, progression-free survival (PFS), and (4) incidence of adverse events.

#### Exploratory endpoints

This study will also explore: (1) the distribution of HRR, MSI, MMR, CDK12 genes mutation and TMB level in locally advanced PCa and OMPCa, and (2) the correlation between the changes of PSMA PET/CT SUVmax values before and after patient-tailored NT and tumor pathological and survival prognosis.

### Determination of sample size

A baseline pCR rate of 5% was estimated based on historical data from neoadjuvant ADT or ADT combined with ARSIs, which have reported pCR rates ranging from 0 to 13% [26]. We hypothesized that a genomic biomarker-guided neoadjuvant regimen would increase the overall pCR rate by an additional 15%, giving a target pCR rate of 20%. Using a one-sided Fisher’s exact test at an 𝛼 level of 0.05 and a power of 80%, we calculated a required sample size of 40 patients.

### Statistical analysis

For categorical variables, frequencies and proportions will be calculated, while for continuous variables, the number of participants, mean, standard deviation, minimum value, median, and maximum value will be calculated. For statistical analysis, categorical variables will be compared with the Chi-square test, and continuous variables will be compared with t-tests. In the survival analyses, Kaplan-Meier survival analyses and log-rank tests will be performed to evaluate PFS and OS. SPSS 22.0 will be used for all statistical analyses. In all tests, *P* < 0.05 will be considered to indicate significance.

### Data management and monitoring

All the completed documents and the informed consent forms will be stored at the study site. Deidentified data collected as part of the study to ensure the confidentiality of the participant. The Institutional Review Board of First Affiliated Hospital of Xiamen University will review and monitor the quality of collected data.

### Patient and public involvement

The patients and public are not involved in the design and conduct of the study.

### Ethics and dissemination

The protocol of this study has been approved by the Institutional Ethics Committee of The First Affiliated Hospital of Xiamen University (XMYY-2024KY040). The study has been registered at ClinicalTrials.gov (NCT06387056). All participants will have to sign and date an informed consent form. The results of this study will be published in peer-reviewed journals and presented at relevant medical conferences. The datasets during the current study are available from the corresponding author on reasonable request.

## Discussion and conclusion

This presented prospective phase II umbrella trial with 4 targeted interventions, to the best of our knowledge, evaluated for the first time whether genomic biomarker-guided NT for locally advanced PCa and OMPCa can result in an improvement in the pathological responses and survival outcomes in a Chinese population. If proven effective, this study will provide high-level evidence to support the implementation of genomic biomarker-guided neoadjuvant therapy for locally advanced PCa and OMPCa. However, as a limitation, it is a single-center, nonrandomized, open-label study. This investigation is currently recruiting participants. This investigation opened the recruitment in China on 1st April, 2024 and the first participant was enrolled on 15th April, 2024.

## Electronic supplementary material

Below is the link to the electronic supplementary material.

Supplementary Box 1

## Data Availability

No datasets were generated or analysed during the current study. The dataset of this study will be available from the corresponding author on reasonable request after completion

## Abbreviations

ADT: Androgen deprivation therapy
AR: Androgen receptor
ARSIs: Androgen receptor signaling inhibitors
cCR: Clinical complete response
dMMR: Mismatch repair–deficient
HRR: Homologous recombination repair
MSI-H: Microsatellite instability-high
NCHT: Neoadjuvant chemo-hormonal therapy
NHT: Neoadjuvant hormonal therapy
NGS: Next-generation sequencing
NT: Neoadjuvant therapy
OMPCa: Oligometastatic prostate cancer
OS: Overall survival
PCa: Prostate cancer
pCR: Pathological complete response
PFS: Progression-free survival
PLND: Pelvic lymph node dissection
pMRD: Pathological minimal residual disease
PSMA PET/CT: Prostate-specific membrane antigen positron emission tomography/ computed tomography
RP: Radical prostatectomy
TMB: Tumor mutational burden
