# Supplementary material for: Safety and efficacy of genomic biomarker-guided neoadjuvant therapy for locally advanced and oligometastatic prostate cancer (SEGNO): study protocol for an open-label prospective phase II umbrella clinical trial
Source: BMC Cancer. 2025 Mar 10;25:432. doi: 10.1186/s12885-025-13826-5 (PMC11895121; doi:10.1186/s12885-025-13826-5)
Supplement: Supplementary file 1 — Supplementary Box 1 [file 12885_2025_13826_MOESM1_ESM.docx]

Inclusion Criteria:

 I. Males ≥ 18 years of age

II. Participant consent must be appropriately obtained in accordance with applicable local and regulatory requirements. Each participant must sign a consent form prior to enrolment in the trial to document their willingness to participate.

III. Imaging examination (defined by multiparametric magnetic resonance imaging, radionuclide bone scan and PSMA PET/CT (prostate-specific membrane antigen positron emission tomography/computed tomography)) confirmed locally advanced (identified as cT3b to cT4, N0 to N1, M0) and oligometastatic (identified as no visceral metastasis and ≤5 bone metastases) prostate cancer. Participants are considered tolerable to radical prostatectomy (RP) and pelvic lymph node dissection (PLND) after neoadjuvant therapy (NT). Participants must consent to RP and PLND after NT when defined as tolerable for RP and PLND at registration and prior to enrolment in the trial.

IV. Histologically confirmed adenocarcinoma of the prostate without pathologic evidence of small cell and neuroendocrine differentiation at the time of initial diagnosis.

V. Eastern Cooperative Oncology Group (ECOG) performance status 0 or 1 and a life expectancy of ≥ 3 years.

VI. Participants must have adequate end-organ function to tolerate NT and subsequent RP plus PLND and all laboratory tests must be performed within 4 weeks prior to registration into master protocol. Including the following indicators: hemoglobin (Hb) ≥85g/L; White blood cell count (WBC) ≥3.0×109 /L; Platelet (PLT) ≥ 75×109 /L; Liver function: Total bilirubin (TBIL) ≤1.5×ULN; Alanine aminotransferase (ALT) and aspartate aminotransferase (AST) ≤1.5× upper limit of normal value (ULN); Albumin (ALB) ≥25g/L; Renal function: glomerular filtration rate (GFR) ≥ 60ml/min.

VII. Participants must consent to genetic testing at registration and prior to enrolment in the trial.

VIII. No prior systemic or localized treatment for prostate cancer. Up to 28 days of Luteinizing hormone releasing hormone agonists (LHRHa), non-steroidal anti-androgen (NSAA) and novel androgen receptor signaling inhibitors (rezvilutamide) are allowable prior to treatment.

IX. Participants must have the ability to swallow oral medication and follow the study procedure.

X. Participants must consent to use reliable contraceptive methods (such as condoms) and not to donate sperm throughout the study period and for 3 months after the last NT administration.

XI. Participants must have no contraindications to any of the relevant drug treatments in the study.

Exclusion Criteria:

 I. Participants with a history of hypersensitivity to any of the relevant drugs involved in this study.

II. Participants received more than 28 days of LHRHa, NSAA and novel androgen receptor signaling inhibitors (rezvilutamide) prior to registration, or enrolled in any other clinical studies for therapeutic purposes within 28 days prior to enrollment, or received any approved anticancer therapy within 28 days prior to enrollment.

III. Participants received local treatments of primary or metastatic lesions prior to enrollment.

IV. Participants received bilateral orchiectomy prior to enrollment.

V. Hypogonadism or severe androgen deficiency as defined by screening serum testosterone more than 50 ng/dL below the normal range for the institution.

VI. Participants with a history of brain metastases or epilepsy.

VII. Participants with severe cardiovascular disease, including: myocardial infarction or thrombosis in the previous 6 months; known unstable angina; history of documented congestive heart failure (New York Heart Association functional classification III-IV; QT interval > 480 ms; uncontrolled hypertension defined as resting systolic blood pressure > 170 mmHg or diastolic blood pressure > 105 mmHg.

VIII. Participants with clinically significant digestive tract abnormalities that may affect the process of drug intake, transport, or absorption (e.g. inability to swallow, chronic diarrhea, intestinal obstruction, etc., or total gastrectomy).

IX. Participants with other clinically significant co-morbidities evaluated by the investigator, including uncontrolled lung disease, active central nervous system disease, active or uncontrolled bacterial, viral, or fungal infections requiring systemic treatment, or any other disease that may make the participant inappropriate for enrollment or RT + PLND after NT.

X. Participants with other known active cancers requiring treatment at the time of entry to the study, or had other malignancies within 5 years prior to enrollment.

XI. Participants received surgeries other than diagnostic prostate biopsy within 4 weeks before enrollment.

XII. Participants with active or known human immunodeficiency virus (HIV) with detectable viral load; active hepatitis B, defined as positive HBV DNA viral load or as defined by relevant guidelines; hepatitis C virus (HCV), except for those have been treated and have an undetectable viral load.

XIII. Participants with a history of non-compliance to medical regimen or inappropriate for the study, attributed to not meeting the principle of optimal benefit treatment.
